# An Atypical Cause of a Child Limp: A Gorham-Stout Disease with a Vanishing Hip

**DOI:** 10.1055/s-0042-1757962

**Published:** 2023-07-31

**Authors:** Hanene Lassoued Ferjani, Yasmin Makhlouf, Dorra Ben Nessib, Kaouther Maatallah, Dhia Kaffel, Wafa Hamdi

**Affiliations:** 1 Departamento de Reumatologia, Kassab Orthopedics Institute, Ksar Saïd, Tunisia

**Keywords:** child, Gorham-Stout disease, hip

## Abstract

Gorham-Stout disease (GSD) is a rare bone disease characterized by an abnormal proliferation of endothelial-lined vessels and destruction of the affected bone. As it affects commonly children and young adults, it is associated with significant morbidity and mortality. To date, there is no established treatment strategy for GSD.

We report through this observation a rare case of GSD in a child located in the hip and the iliac crest.

## Introduction

Gorham-Stout disease (GSD) or ‘vanishing bone’ is a rare bone disease of unknown etiology. 1 The involved mechanism is osteolysis through the proliferation of the vascular structures of the bone tissue, resulting in the destruction of the bone matrix with fibrosis. 1 The pathologic process consists of the replacement of normal bone by an aggressive expanding vascular tissue. 2 The first case was described in 1838 and involved the humerus. Since then, only 300 cases have been reported in the literature, and no association with genetic or immunological factors has been established. 3 Gorham-Stout disease may develop in any skeleton site, but it commonly affects the shoulder, the pelvic girdle, and the skull, rarely the femur. 4 This vanishing bone disease can be severely disabling, and ∼ 16% of the cases are fatal. 3 Nevertheless, the diagnosis of GSD should be suspected only after excluding other causes of osteolysis.

Herein, we describe a case of GSD involving the femur and the right iliac crest in a child.

## Clinical Case

An 11-year-old boy presented with a limp in his right leg and used two canes to walk. There was no family history of any bone- or joint-related problems. His medical history dates to 1 year ago when he suffered from painless limping without fever. The patient did not experience lethargy or fatigue. He was diagnosed with pathological fracture and treated with drilling and curettage with no postoperative complication. At that time, the histopathological findings were not conclusive. At follow-up, the patient did not report improvement after operative treatment.

On physical examination, the incision scar was clean, and the right thigh was swollen. We noted a decreased range of motion, in comparison with the uninvolved hip, with respectively: flexion to 90°, extension to 10°, abduction to 30°, adduction to 30°, internal rotation to 10, and external rotation to 20°.

**Fig. 1:**
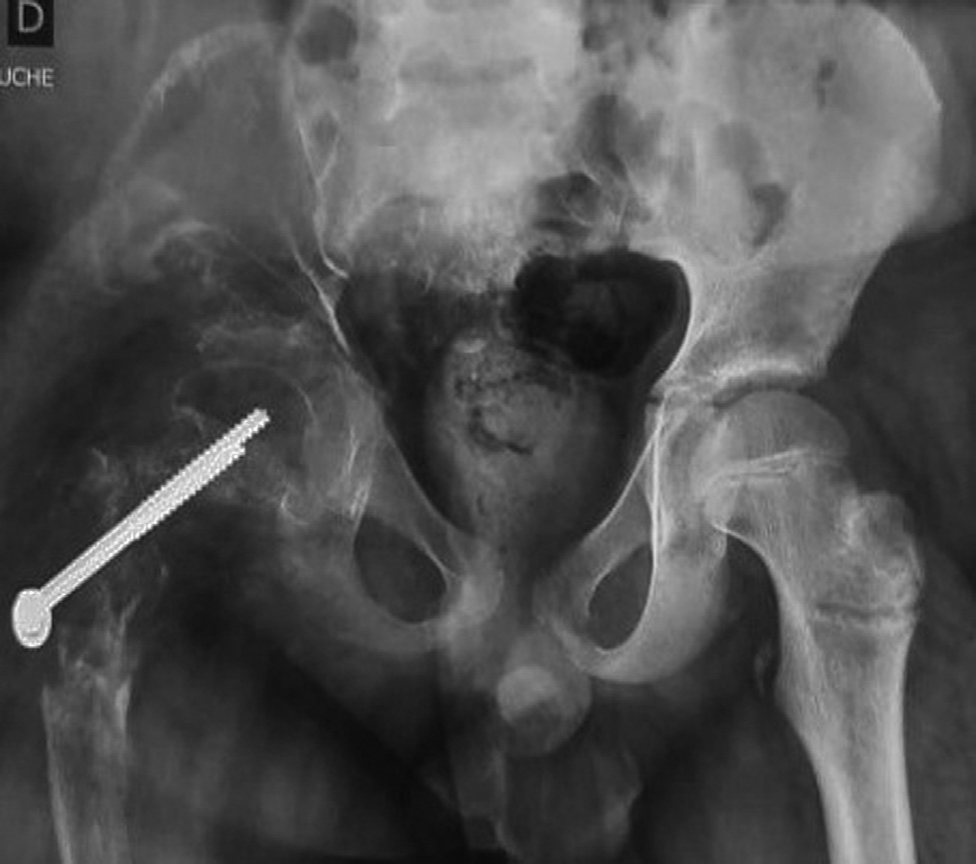
X-ray of the pelvis showing massive osteolysis across the right proximal femur and the iliac crest.

**Fig. 2:**
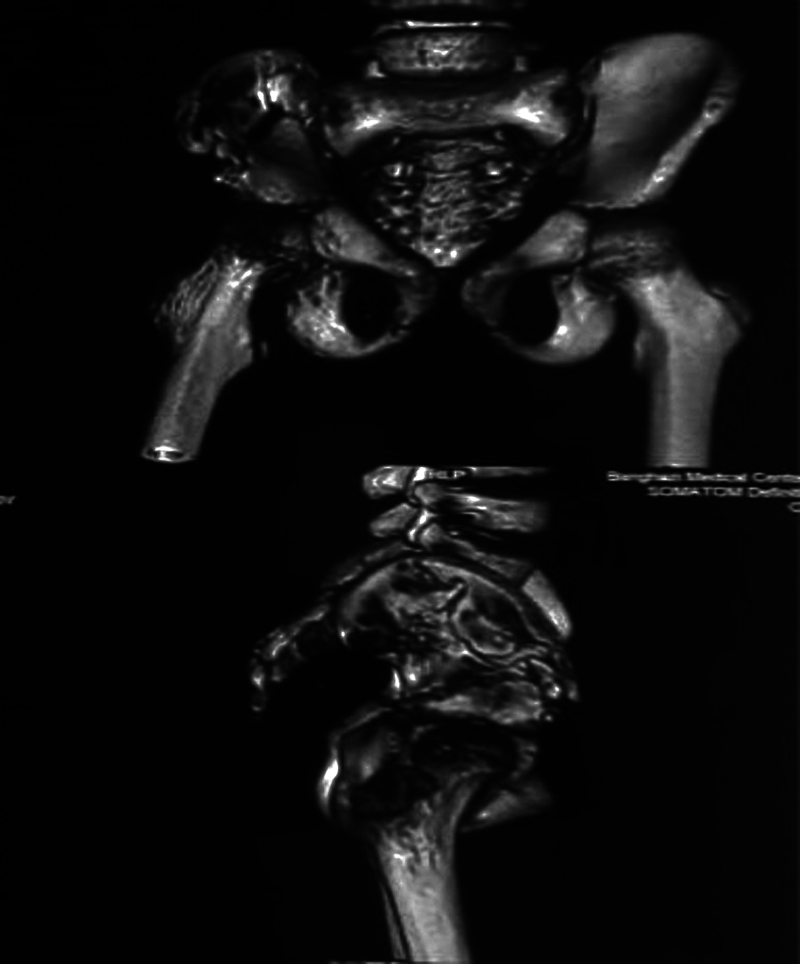
Computed tomography 3D reconstruction in a child with GSD showing osteolysis of the right proximal femur and iliac crest.

The pelvis radiograph and computed tomography (CT) revealed an osteolytic lesion with multiple bony fragments across the iliac crest and the proximal femur ( Figs. 1 , 2 ). The laboratory test results were in the normal range. The erythrocyte sedimentation rate (ESR) and C-reactive protein (CRP) were at 20 mm/h and 3.9 mg/L, respectively.

**Fig. 3:**
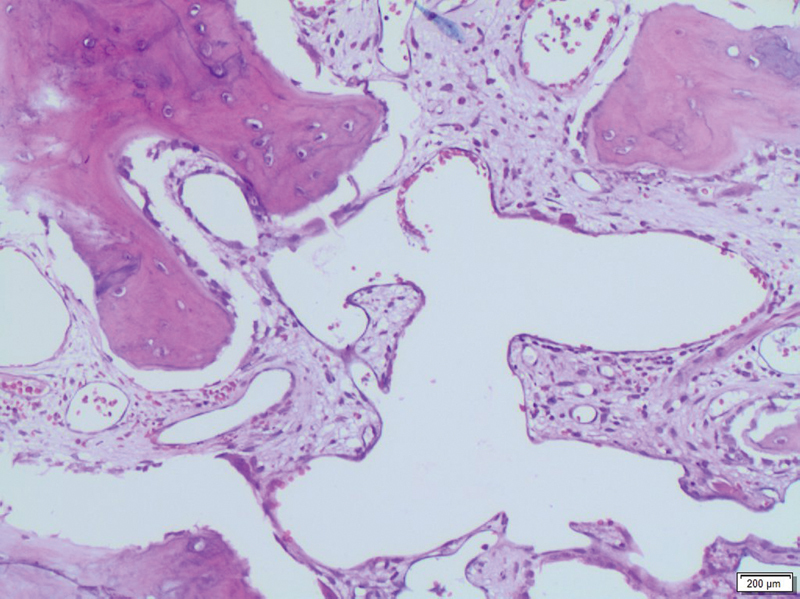
Histological image showing bone remodeling with foamy macrophages, and the hematic and lymphatic vascular channels proliferation (hematoxylin-eosin, original magnification × 200).

To rule out the malignant tumor, the patient underwent a whole-body CT. The latter did not reveal any primary tumor or lytic lesions located in other sites. A bone marrow biopsy of the iliac crest ruled out chronic infection and malignancies. The histopathological exam showed bone remodeling with foamy macrophages, and the proliferation of hematic and lymphatic vascular channels confirming the diagnosis of GSD ( Fig. 3 ).

## Discussion

We report a rare case of GSD in a child, located in the femur and the iliac crest and revealed with painless limp. Gorham-Stout disease, known as the ‘vanishing bone disease,’ is a rare form of massive spontaneous or progressive osteolysis not followed by new bone production. 5 Gorham-Stout disease was first described in 1838, reporting a case of a disappearing humerus in a young man. 6 Since then, due to the low incidence of GSD, the current literature was confined to reports and case series (∼ 300). 3

The disease may present at any age without gender predilection but targets mainly young adults and children as in our report. 6 One of the proposed mechanisms is an uncontrolled lymphangiogenesis driven by the vascular endothelial growth factor (VEGF) with the mammalian target of rapamycin (mTOR) expression. 7 This local proliferation is responsible for bone loss around the lymphatic vessels. 6 In addition, there is an increase in osteoclast activity as evidenced by the high osteoclast precursor activity and the increased level of IL6. 6

From a clinical point of view, single-centered bone is usually affected. 8 The most commonly involved sites are the mandible (15%), the scapula (10%), the ribs (12%), the humerus (8%), the pelvis (10%), and the femur (11%). 3 Spine and thorax involvement may lead to paraplegia and respiratory complications. 9 Similar to our case, the disease is progressive and the diagnosis is delayed because of the low intensity of pain. In agreement with the reported data, the pathological fracture is the first disease presentation as in the current case in which imaging findings showed large bone osteolysis without evidence of repair. While radiological features were overlapping with the neoplastic lesion and endocrine disorders in the present report, the biological and histological results supported the GSD diagnosis in the pelvis and the proximal femur.

Regarding treatment modalities, there are no Food and Drug Administration-approved therapies for treating GSD. 10 Surgery is usually the first-line treatment and consists of resection followed by reconstruction or endoprosthetic replacement in weight-bearing bones. If the latter is not possible, medications might be effective. The medical treatment included sirolimus, or bisphosphonate, and Interferon α-2b (IFNα-2b). 10 The sirolimus, an mTOR- inhibitor, acts as a downregulator of cellular proliferation without adverse effects on normal lymphatics. The IFNα-2b, used in single or in combination therapy with bisphosphonates showed an encouraging result for a long-term follow-up. 10

Radiotherapy is also another alternative. However, considering the young age of our patient and the late effects on the pelvic organ and growth, we preferred to avoid radiotherapy. 3 Unfortunately, we did not have the possibility to prescribe the IFN or sirolimus in our case because of the high cost. The patient was treated only with bisphosphonate (Zoledronic acid).

The lack of trials has hindered the identification of an effective strategy to adopt for treating this disease. More importantly, several studies are underway in the hope of unraveling the mystery of this disease and addressing unresolved questions.

We report an exceptional case of GSD in a child located in the hip. Given the aggressiveness of the lesion, clinicians should be aware of this rare disease for prompt treatment and better management.
