# Supplementary material for: An Atypical Cause of a Child Limp: A Gorham-Stout Disease with a Vanishing Hip
Source: Rev Bras Ortop (Sao Paulo). 2023 Jul 31;59(Suppl 1):e101–3. doi: 10.1055/s-0042-1757962 (PMC11254440; doi:10.1055/s-0042-1757962)
Supplement: Related texts

# Research article: Uma causa atípica de claudicação infantil: Síndrome de Gorham-Stout com quadril fantasma

**Keywords:** criança, quadril, síndrome de Gorham-Stout

## Abstract

A síndrome de Gorham-Stout (SGS) é uma doença óssea rara caracterizada pela proliferação anormal de vasos endoteliais e destruição do osso acometido. Por ser comum em crianças e adultos jovens, causa morbidade e mortalidade significativas. Até o momento, não há estratégia terapêutica estabelecida para a SGS.

Relatamos um caso raro de SGS no quadril e na crista ilíaca de uma criança.

## Introdução

A síndrome de Gorham-Stout (SGS) ou “doença do osso fantasma” é uma doença óssea rara de etiologia desconhecida. 1 É causada por osteólise e proliferação de estruturas vasculares do tecido ósseo, o que provoca destruição da matriz óssea e fibrose. 1 O processo patológico consiste na substituição do osso normal por um tecido vascular agressivo em expansão. 2 O primeiro caso foi descrito em 1838, no úmero. Desde então, apenas 300 casos foram relatados na literatura, sem estabelecimento de nenhuma associação a fatores genéticos ou imunológicos. 3 A SGS pode ser observada em qualquer parte do esqueleto, mas é mais comum no ombro, na cintura pélvica, no crânio e, raramente, no fêmur. 4 Esta doença osteolítica pode causar incapacidade grave e ∼ 16% dos casos são fatais. 3 No entanto, deve-se suspeitar de SGS somente após a exclusão de outras causas de osteólise.

Aqui, descrevemos um caso de SGS no fêmur e na crista ilíaca direita de uma criança.

## Caso Clínico

Um menino de 11 anos apresentou claudicação do membro inferior direito e usava duas bengalas para andar. Não havia histórico familiar de problemas ósseos ou articulares. Seu histórico médico data de 1 ano atrás, quando o paciente apresentava claudicação sem dor ou febre. O paciente não tinha letargia ou fadiga. Foi diagnosticado com fratura patológica e tratado com perfuração e curetagem sem intercorrências pós-operatórias. Naquela época, os achados histopatológicos não eram conclusivos. No acompanhamento, o paciente não relatou melhora após o tratamento cirúrgico.

Ao exame físico, a cicatriz da incisão estava limpa e a coxa direita edemaciada. Observamos uma diminuição da amplitude de movimento em comparação ao quadril não acometido de 90° de flexão, 10° de extensão, 30° de abdução, 30° de adução, 10° de rotação interna e 20° de rotação externa.

A radiografia e a tomografia computadorizada (TC) da pelve revelaram uma lesão osteolítica com múltiplos fragmentos ósseos na crista ilíaca e no fêmur proximal ( Figs. 1 , 2 ). Os resultados dos exames laboratoriais estavam dentro da faixa de referência. A velocidade de hemossedimentação (VHS) e a concentração de proteína C reativa (PCR) foram de 20 mm/h e 3,9 mg/L, respectivamente.

**Fig. 1:** Radiografia da pelve revela osteólise maciça em todo o fêmur proximal e crista ilíaca do lado direito.

**Fig. 2:** Reconstrução tridimensional da tomografia computadorizada de uma criança com síndrome de Gorham-Stout mostra a osteólise do fêmur proximal e da crista ilíaca do lado direito.

Para descartar a presença de um tumor maligno, o paciente foi submetido a uma TC de corpo inteiro. Este exame não revelou nenhum tumor primário ou lesões líticas em outros sítios. A biópsia da medula óssea da crista ilíaca descartou infecção crônica e câncer. O exame histopatológico revelou remodelação óssea com macrófagos xantomatosos e proliferação de canais vasculares hemáticos e linfáticos, confirmando o diagnóstico de SGS ( Fig. 3 ).

**Fig. 3:** A imagem histológica mostra a remodelação óssea com macrófagos xantomatosos e proliferação de canais vasculares hemáticos e linfáticos (hematoxilina-eosina, aumento original × 200).

## Discussão

Relatamos um caso raro de SGS localizada no fêmur e na crista ilíaca de uma criança e revelado por claudicação indolor. A SGS, conhecida como “doença do osso fantasma”, é uma forma rara de osteólise maciça espontânea ou progressiva não acompanhada por produção de novo tecido ósseo. 5 A SGS foi descrita em 1838, com relato de um caso de desaparecimento do úmero em um homem jovem. 6 Desde então, devido à baixa incidência da síndrome, a literatura é limitada a relatos e séries de casos (quase 300). 3

A doença pode surgir em qualquer idade e não tem predileção por sexo, mas é observada principalmente em adultos jovens e crianças, como em nosso relato. 6 Um dos mecanismos propostos é uma linfangiogênese descontrolada impulsionada pelo fator de crescimento endotelial vascular (VEGF) e expressão do alvo mamífero de rapamicina (mTOR, na sigla em inglês). 7 Esta proliferação local é responsável pela perda óssea ao redor dos vasos linfáticos. 6 Além disso, há um aumento na atividade dos osteoclastos, evidenciado pela alta atividade precursora dos osteoclastos e pelo aumento do nível de interleucina 6 (IL-6). 6

Do ponto de vista clínico, um único centro ósseo é geralmente acometido. 8 Os sítios mais comuns de SGS são a mandíbula (15%), a escápula (10%), as costelas (12%), o úmero (8%), a pelve (10 %) e o fêmur (11%). 3 O acometimento da coluna e do tórax pode provocar paraplegia e complicações respiratórias. 9 Como em nosso caso, a doença é progressiva e o diagnóstico é tardio devido à baixa intensidade da dor. De acordo com os dados relatados, a fratura patológica é o primeiro sintoma da doença, como aqui, em que as técnicas de diagnóstico por imagem mostraram grande osteólise óssea sem evidência de reparo. Embora as características radiológicas tenham se sobreposto à lesão neoplásica e distúrbios endócrinos neste quadro, os resultados biológicos e histológicos apoiaram o diagnóstico de SGS na pelve e no fêmur proximal.

Em relação às modalidades para o tratamento da SGS, não há nenhuma aprovada pela Food and Drug Administration (FDA, na sigla em inglês) dos Estados Unidos. 10 De modo geral, a cirurgia é o tratamento de primeira linha e consiste na ressecção seguida de reconstrução ou substituição endoprotética de ossos de sustentação de peso. Se isso não for possível, alguns medicamentos poderiam ser eficazes. O tratamento médico incluiu sirolimus ou bifosfonato e interferon α-2b (IFNα-2b). 10 O sirolimus, o inibidor de mTOR, atua como um regulador negativo da proliferação celular sem efeitos adversos sobre os vasos linfáticos normais. O IFNα-2b, como terapia única ou combinado a bisfosfonatos, teve resultados animadores no acompanhamento em longo prazo. 10

A radioterapia também é uma alternativa. No entanto, considerando a pouca idade do nosso paciente e os efeitos tardios nos órgãos pélvicos e no crescimento, preferimos evitá-la. 3 Infelizmente, não pudemos prescrever IFN ou sirolimus no nosso caso devido ao alto custo. O paciente foi tratado apenas com bifosfonato (ácido zoledrônico).

A ausência de ensaios tem dificultado a identificação de uma estratégia eficaz para o tratamento da SGS. É importante ressaltar que vários estudos estão em andamento na esperança de desvendar os mistérios desta doença e abordar questões não resolvidas.

Relatamos um caso excepcional de SGS no quadril de uma criança. Devido à agressividade da lesão, os médicos devem estar atentos a esta rara doença para tratamento imediato e melhor manejo.
